# Developing a workplace lactation promotion model in Indonesia using Delphi technique

**DOI:** 10.1186/s13690-018-0312-2

**Published:** 2018-11-05

**Authors:** Ray Wagiu Basrowi, Sudigdo Sastroasmoro, Astrid W Sulistomo, Saptawati Bardosono, Aryono Hendarto, Dewi S Soemarko, Ali Sungkar, Levina Chandra Khoe, Yvan Vandenplas

**Affiliations:** 1 Doctoral Program Student, Faculty of Medicine, Universitas Indonesia, Jakarta, Indonesia; 2 Department of Child Health, Faculty of Medicine, Universitas Indonesia, Jakarta, Indonesia; 3 Department of Community Medicine, Faculty of Medicine, Universitas Indonesia, Jakarta, Indonesia; 4 Department of Nutrition, Faculty of Medicine, Universitas Indonesia, Jakarta, Indonesia; 5 Department of Obstetric Gynecology, Faculty of Medicine, Universitas Indonesia, Jakarta, Indonesia; 6 KidZ Health Castle, UZ Brussel, Vrije Universiteit Brussel, Laarbeeklaan 101, 1090 Brussels, Belgium

**Keywords:** Breastfeeding, Lactation, Women worker, Workplace lactation promotion

## Abstract

**Background:**

Working mothers have a higher risk to terminate breastfeeding earlier than stay-at-home mothers. Researchers reported that support from the workplace by creating lactation facilities and develop supportive programs are necessary to increase the success of exclusive breastfeeding. The aim was to achieve expert consensus on developing a workplace-based lactation promotion model.

**Methods:**

A three-round online survey using Delphi approach was conducted to reach consensus on to the development of a lactation program at a workplace.

**Results:**

Twenty-two experts from Indonesian health authority, community medicine, child health and obstetrics were invited to join the Delphi study; 15 (68.2%) enrolled in the first round. The response rate in the second and third round was 80.0% (12/15) and 86.7% (13/15), respectively. The first round categorized the workplace-based promotion model into seven dimensions, i.e. policy and regulation, facility, education material, target participants, promotion approach, human resources, and time. In the final round, “maternity leave of 3-6 months” (median (Q1;Q3):2 (1, 4)) and “employees have the right to breast-pumping every 3 hours” (median (Q1;Q3):3 (2, 4)) ranked as the two most important indicators regarding policy and regulation. A dedicated lactation room (median (Q1;Q3):1 (1)) is the highest ranked indicator regarding facility dimension. Regarding education materials, benefits of breast milk for babies ranked as the highest indicator while for the education and delivering methods dimensions, social media and interactive counseling were two highest ranked indicators. The top management in the company and lactation counselor are the two highest-ranked indicators in human resources dimension.

**Conclusion:**

In the view of experts, involvement of a dedicated policy maker in the company, a workplace-based lactation counselor, regular promotion with interactive education and dedicated facilities are necessary to develop an effective workplace-based lactation promotion model.

## Background

Nowadays, more women are working out-house than ever before. This phenomenon occurs not only in industrialized countries, but also in developing countries, such as Indonesia. The National Statistic Agency (BPS) noted a-fourfold increase of women labor force in 2014 compared to 2008. This increasing trend of female workers has an important impact on their role as mother, especially for breastfeeding mothers. Breastfeeding yields significant health benefits for mothers and their child that extend into adulthood. Breastfeeding reduces the risk of breast and ovarian cancer, risk of type 2 diabetes, and increases the duration of lactational amenorrhea [1]. It is also associated with a protective effect on diarrhea and respiratory infections and a lower risk of type 2 diabetes in adulthood [2]. WHO and UNICEF has globally recommended exclusive breastfeeding as the most ideal food for infants in their first six months of life [3].

The government of Indonesia set a national target of 80% exclusive breastfeeding. Unfortunately, Indonesia fails to reach the target as only 30,2% was achieved [4]. Many factors influence the decision of a mother to breastfeed or not, including breast problems, birth delivery method, education level, support from husband and family, and employment status [5–9]. The exclusive breastfeeding rate among female workers in Indonesia was very low, only 19%. Employed mothers have higher risk to terminate breastfeeding earlier than stay-at-home mothers [10].

This study explored the opinion of Indonesian experts on elements contributing to breastfeeding promotion in working places, using the Delphi technique. The Delphi method is a structured communication process and comprises several rounds of surveys. It is commonly used as a method for consensus building. In this study, we aimed to achieve a consensus on the most needed actions to promote lactation at the workplace.

## Methods

**Fig. 1:**
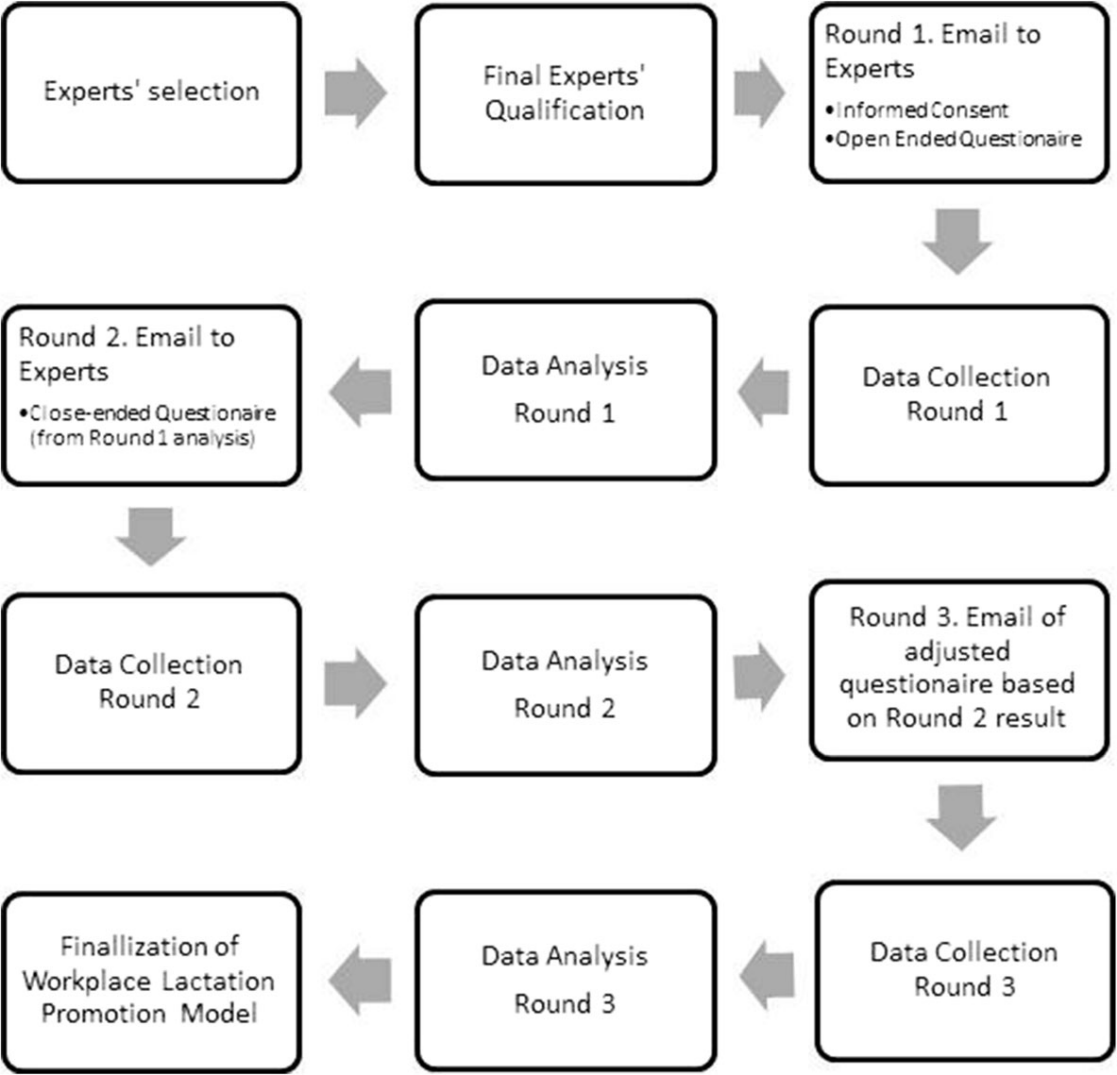
Flow of the Delphi Study (Indonesia, November 2016–May 2017, Workplace lactation model)

A three-round online survey (Fig. 1) using Delphi approach was conducted between November 2016 to May 2017 to explore the opinion of experts on issues related to a lactation program and support at the workplace.

In the first round, open-ended questions were asked, which included policy and regulation, facilities, education materials, target participants, promotion methods, time, and human resources. Two government policy documents in the field of lactation support in workplace, i.e. (1) Joint Ministerial Regulations between the Minister of Women Empowerment, the Minister of Labor and Transmigration, and the Minister of Health in 2008 about the improvement in providing breastfeeding support during work time at workplace, and (2) the Minister of Health Regulation No 15 year 2013 about the Procedure on the provision of special facilities for breast milk, were used as the main reference. The results of the first round were used to develop the second round questionnaire. In the third round, results from the second round were presented.

All communication occurred by e-mail. During every round, several reminders by emails, mobile messages, and telephones were sent out to promote participation. The study was approved by The Ethical Committee of Faculty of Medicine Universitas Indonesia letter number 786/UN2.F1/ETIK/2016.

### Selection of experts

The experts were selected based on their academic qualifications and working experiences related to the lactation program at the workplace. The participants were invited as experts and selected from the database from the Community Medicine Department, Faculty of Medicine Universitas Indonesia and were selected from the following different areas of expertise: occupational medicine, occupational health, pediatrics, obstetrics and government (Table 1).

**Table 1:** Characteristics of the experts participating in the Delphi rounds (Indonesia, November 2016–May 2017, Workplace lactation model)

Variables	Round 1 *n* = 15					Round 2 *n* = 12					Round 3 *n* = 13				
	occupational medicine	occupational health	pediatrics	obstetric	government	occupational medicine	occupational health	pediatrics	obstetric	government	occupational medicine	occupational health	pediatrics	obstetric	government
Gender															
male	2		1	1	1	2		1	1		2		1	1	1
female	1	3	4	1	1	1	3	2	1	1	1	3	2	1	1
Age															
40–49 years	3	1	2	2	1	3	1	2	2	1	3	1	1	2	1
50–59 years		2	1		1		2					2	1		1
> 60 years			2					1					1		
Length of career															
< 10 years	2	1	1	2	1	2	1	1	2	1	2	1	1	2	1
10–14 years	1	1	1			1	1	1			1	1	1		
15–19 years			1		1										1
> 20 years		1	2				1	1				1	1		

### Analysis

Content analysis was performed in the first round of Delphi to group and categorize the answers from all experts, based on the similarity of keywords, phrases and themes derived from the open-ended questionnaire. The ranked-based indicators from the second and third round were further analyzed using SPSS version 20 to calculate the median and interquartile range in order to gives a better indication of the experts’ consensus on each of the items in the questionnaire. All of the items indicate in the 75% above (Q4) are indicated as the least frequent selected items from experts, therefore less important and hence excluded. Only items falling between quartile-1 (Q1) to quartile-3 (Q3) were further included into the final model. The median value was calculated to identify the ranking of each set of the indicators.

### Round 1

The aim of the first round was to gather the opinion of the experts through open-ended questions. Information about this research and an informed consent were sent via e-mail before the 1st round questions were distributed. The questions were send in Microsoft Word format. The participants could type the answers and return the response by e-mail. For each question, answers with similar keywords/themes were classified in the same group. A set of indicators was developed based on the answers (Table 2).

**Table 2:** The seven major aspects of the lactation promotion model at the workplace (Indonesia, November 2016–May 2017, Workplace lactation model)

1. *Company’s policy and regulation* indicated Employees have right to breast pumping every 3 h; Maternity leave for 3–6 months, Employees have right to choose to be part-time worker during the first 6-months after delivery; Employees could return home earlier during the first 6-months after delivery; Employee who does exclusive breastfeeding will be given award; Providing regular lactation education; Limiting extra work/out of town work for breastfeeding employees; Providing extra food for breastfeeding employees; Providing special sign (priority) for breastfeeding employees 2. *Facility* indicated Fridge; Dedicated lactation room; Comfortable chair; Sink; Breastmilk pump; Breastmilk delivery service from work to home; Bottle milk sterilizer; Cooler bag; Water dispenser; Child-care service; Health magazine and Posters 3. *Education Materials* indicated Benefit of breastmilk for babies; Procedure of breastmilk pumping; Methods to store breastmilk; Methods to maintain the breastmilk hygiene; Breast care; Nutrition for breastfeeding mothers; Physiology of lactation; Benefit of early breastfeeding; Support from working environment; Benefit of breastmilk for mothers; Benefit of breastmilk for workers; Family planning; Effect of stress on lactation behavior; Challenges for breastmilk 4. *Target Participants* indicated All employees return from maternity leave; All employees who breastfeed after return from maternity leave; All women employees (including unmarried); All women employees in productive age (18–50 years); All pregnant employees; All pregnant employees with gestational age ≥ 32 weeks; All male employees whose wifes are pregnant; All male employees whose wifes breastfeeding 5. *Education Method* indicated Interactive counseling/lecture; Social media; Poster; Private counseling; Movie/video; Demonstration; Leaflet, Group discussion 6. *Time* indicated ±15 (fifteen) minutes to 1 (one) hour face-to-face discussion/interactive counseling and/or lecture and/or watching educational movies during lunch break or breast-pumping break; direct short-message or whatsapp message via social media group or Whatsapp/broadcast message/email everyday/at least once a week. 7. *Human Resources* indicated Top management of the company; Lactation counselors; Human Resources Department; Company’s doctor; Labor union; Colleagues/peers	

### Round 2

The feedback from the 1st round was informed in the narrative form before the 2nd round questionnaires. The second round was composed of 7 main topics. In each topic, there were a number of indicators. The participants were asked to rank the indicators based on the estimated importance, “number 1” indicating the most important. A column of reasoning was provided in case the participants would like to add some comments to the indicators.

### Round 3

The response from the 2nd round was presented in a median and interquartile range to each expert. Indicators ranked beyond 75th percentile (least important) were excluded. The participants were also informed about the excluded indicators. A same set of questions was distributed to the same participants invited in the 2nd round. The results obtained from the 3rd round (Table 3) were further evaluated and compared with the 2nd round.

**Table 3:** Descriptive statistics for the final recommendation of a workplace-based lactation promotion model (Indonesia, November 2016–May 2017, Workplace lactation model)

Dimensions	Item Indicators		Round 2 Median (Q1;Q3)	Round 3 Median (Q1;Q3)
1 Company’s regulation	a	Employees have right to breast pumping every 3-h	4 (2;7)	3 (2,4)
	b	Maternity leave for 3–6 months	1,5 (1;2)	2 (1,4)
	c	Employees have right to choose to be part-time worker during the first 6-months after delivery	4 (2;7)	4 (3,5)
	d	Employees could return home earlier during the first 6-months after delivery	4 (3;7)	4 (2,5)
	e	Employee who does exclusive breastfeeding will be given appreciation	7 (6;9) Excluded	
	f	Providing education on lactation regularly	5 (2;7)	3 (2,6)
	g	Limiting extra work/out of town work for breastfeeding employees	5 (5;7)	5 (3,6)
	h	Providing extra food for breastfeeding employees	6 (4;8) Excluded	
	i	Providing special sign (priority) for breastfeeding employees	7 (5;9) Excluded	
2 Facilities	a	Fridge	2 (1;3)	2 (2,3)
	b	Special breastfeeding room	1 (1;2)	1 (1,1)
	c	Comfortable chair	4 (3;6)	4 (2,6)
	d	Sink	3 (3;4)	4 (3,5)
	e	Breastmilk pump	7 (5;8)	6 (5,7)
	f	Breastmilk delivery service from work to home	10 (4;11) Excluded	
	g	Bottle milk sterilizer	6 (5;8)	5 (4,6)
	h	Cooler bag	8 (6;9)	7 (6,8)
	i	Water dispenser	7 (6;9)	6 (7,8)
	j	Childcare service	10 (8;10) Excluded	
	k	Health magazine	9 (9;12) Excluded	
	l	Poster	11 (7;11) Excluded	
3 Education materials	a	Benefit of breastmilk for babies	1 (1;2)	1 (1,2)
	b	Procedure of breastmilk pumping	4 (2,75; 5)	3 (2,4)
	c	Methods to store breastmilk	4 (3; 5,5)	3 (3,4)
	d	Methods to maintain the breastmilk hygiene	5 (4; 6)	4 (4,5)
	e	Breast care	6,5 (5,75; 11,5)	5 (6,9)
	f	Nutrition for breastfeeding mothers	7 (5,5; 8,25)	7 (6,9)
	g	Physiology of lactation	11 (7,75; 14) Excluded	
	h	Benefit of early breastfeeding	8 (2,75; 9,25)	7 (2,9)
	i	Support from working environment	8,5 (2,5; 9)	8 (7,9)
	j	Benefit of breastmilk for mothers	7 (2; 10,5)	7 (5,10)
	k	Benefit of breastmilk for workers	10 (7,75; 11)	8 (4,9)
	l	Family planning	11,5 (10,75; 13) Excluded	
	m	Effect of stress on lactation behavior	11,5 (11,5; 12,25) Excluded	
	n	Challenges for breastmilk	12,5 (10; 14) Excluded	
4 Target Participants	a	All employees return from maternity leave	3,5 (1,25; 5,5)	4 (1,5)
	b	All employees who breastfeed after return from maternity leave	3 (3; 5)	4 (3,5)
	c	All women employees (including unmarried)	6 (2; 8)	6 (4,6)
	d	All women employees in productive age (18–50 years)	4 (2; 5)	3 (1,5)
	e	All pregnant employees	3 (1; 3)	3 (2,3)
	f	All pregnant employees with gestational age ≥ 32 weeks	4 (1; 5)	3 (2,4)
	g	All male employees whose wife’s are pregnant	7 (5; 7) Excluded	
	h	All male employees whose wife’s breastfeeding	7 (6; 8) Excluded	
5 Education methods	a	Interactive lectures	3 (1; 5,5)	3 (1,5)
	b	Social media	3 (2; 5)	2 (1,5)
	c	Poster	5 (3; 7)	5 (4,6)
	d	Private counseling	3 (1; 5)	3 (2,6)
	e	Movie/video	5 (4; 7)	5 (4,5)
	f	Demonstration	6 (4; 6)	4 (3,5)
	g	Leaflet	7 (6; 8) Excluded	
	h	Group discussion	3 (2; 4)	4 (3,7)
6 Human resources	a	Head of companies	2 (1; 2,75)	
	b	Lactation counselors	3 (1; 4)	
	c	Human Resources Department	3 (2; 5)	
	d	Company’s doctor	3 (2; 4)	
	e	Labor union	6 (4; 6) Excluded	
	f	Colleagues / peers	5 (4; 6) Excluded	

## Results

**Fig. 2:**
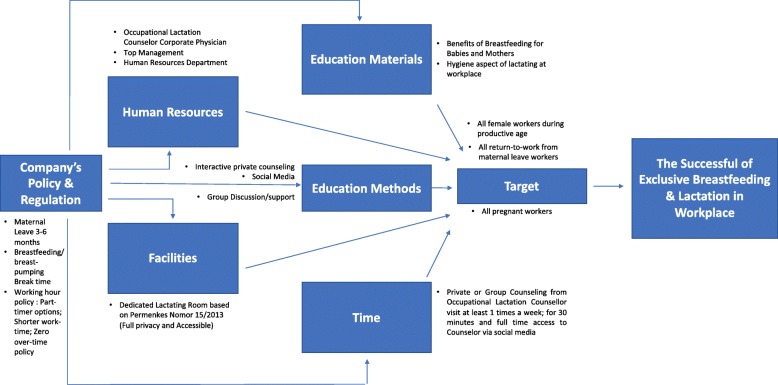
Workplace Lactation Promotion Model (Indonesia, Basrowi et al 2018)

Twenty-two experts were invited to join the Delphi study, but only 15 enrolled (68.2%). In the second round, 80.0% (12/15) participated and 86.7% (13/15) in the third round. The experts consisted of various stakeholders that related to the lactation program at work place. The final result from three rounds was formulized into a set of workplace-based lactation promotion model, i.e. policy and regulation, facility, education material, target participants, promotion approach, human resources and time (Fig. 2).

### Company’s policy & regulation

The first round resulted in 9 items regarding the policy and regulation in the company for lactating female workers. These included the opportunity to pumping breast milk during office hours, duration for maternity leave, part-time policy for return-to-work-mothers, additional nutritious food for breastfeeding-workers, priority signage for breastfeeding-workers, regular health education to support breastfeeding and rewards for female workers who give exclusive breastfeeding. In the second round, three items were excluded. In the final round, maternity leave 3–6 months (Median (Q1;Q3):2 (1, 4)) and employee have right to breast-pumping every 3 h (Median (Q1;Q3):3 (2, 4)) ranked as the two most important indicators for company’s policy and regulation.

### Facility

There were 12 necessary facilities to support breastfeeding mothers at work. Most of them were already listed in the government regulation. Four items were eliminated in the second round, i.e. breast milk delivery service, daycare, health-related magazine, and poster. These excluded items were not mentioned in the existing regulation on breastfeeding. The must-have-items confirmed by the experts in the round three were refrigerator to keep the breast milk (Median (Q1; Q3):2 (2, 3)), a dedicated lactation room (Median (Q1; Q3):1 (1)), and a comfortable chair (Median (Q1; Q3): 4 (2, 6)).

### Education material

The first round yielded fourteen topics for education materials. But in the second round, four were excluded, which were the physiology of lactation, family planning, how stress is affecting lactation behavior, and problems found in breastfeeding. The third round left nine topics related to the benefits and methods of lactation, important nutrition for breastfeeding mothers, breast care, and support from the working environment towards lactating behavior.

### Target participants

In the first round, the experts mentioned that male workers with pregnant or breastfeeding wives should also be targeted in the program. However, when the experts were asked to prioritize, this group was excluded, leaving all female workers as target participants. The last round confirmed women in the productive age (18–50 years old), pregnant workers, and specifically in their third trimester should be included as target participants.

### Education method

Various methods were included in the first round, i.e. interactive lecture, promotion through social media, poster, private counseling, promotion through movie/video, demonstration, leaflets, and group discussion. The second round eliminated the promotion through leaflets. And finally, the third round considered the approach using interactive lecture (Median (Q1;Q3): 3 (1, 5)), social media (Median (Q1; Q3):2 (1, 5)), and private counseling (Median (Q1; Q3):3 (2, 6)) to be prioritized for the breastfeeding program.

### Human resources

The company directors, lactation counselors, the human resource department, company medical doctors, the labor union, and peers were mentioned as important human resources for the breastfeeding program. The second round excluded labor union and peers. Finally, the experts selected top management/company directors, lactation counselors, and company medical doctors as the most significant human resources for the breastfeeding program.

### Time

There were several subtopics discussed under the theme of time. The experts agreed that the lactation counselor should be ready upon the call (on-call duty), and not necessarily need to standby at the company in the office-hour. Regarding the schedule for health education, most experts agreed that it should be held during weekdays, once a week for around 30 min.

## Discussion

As working mothers, support from the workplace is indeed necessary to promote the success of exclusive breastfeeding. The availability of lactation facilities and supportive program at workplace increases lactation practice by three-fold and increases the exclusive breastfeeding rate by six-fold [11]. It is also related with the productivity improvement and lower absenteeism [12]. However, previous study identified only 21.5% of female workers obtained the access of lactation facilities at work and 7.5% of them had lactation support program at their workplace [11]. The existing module for breastfeeding support from the government is targeting general population; and not specific for working mothers. Some adjustments are necessary since the working environment affects the mother’s motivation to breastfeed.

To our best knowledge, this is the first study developing a workplace-based lactation promotion model in Indonesia. Delphi study is a common method in developing the medical curriculum [12–14]. In electronic survey, a minimum response rate of 50% is necessary to obtain a valid result [15]. In this study, each round had response rate more than 50%. It demonstrated that experts put attention on and value the importance of this study.

The government of Indonesia issued several regulations to support breastfeeding program at work place. The Government Regulation No. 33 of 2012 mentioned that employers should support on exclusive breastfeeding program by providing lactation room at workplace. The Minister of Health also issued the Regulation No.15 of 2013 regarding certain private lactation room, and even provides the specific guidelines for breastfeeding facilities. However, not all employers are complied with the regulation. Many working mothers breastfeeding and breast pumping in the toilets or praying room; and keep in the refrigerators along with other foods [10]. The Ministry of Women’s Empowerment, Ministry of Manpower, and Ministry of Health issued a common regulation on breastfeeding during work hours. However, in reality, the labors have certain target in each day, and they have limited time to finish their target. Some working mothers chose not to breastfeed deliberately since it would disturb their work pace.

The policy on having maternity leave more than three months ranked first as expert’s recommendation. Several studies indicated that the duration of maternity leave is an important factor in the sustainability of lactation behavior among female workers. However, experts specifically recommended the workplace regulation on giving permission for breastfed employees to have their breastfed time every 3-h, giving choice as part-timer or return home earlier for workers with under-6-month child. The experts recommended dedicated lactation room with additional facilities, such as cold storage, chairs, water sink, and breast milk pumping. These items were also recommended by the guidelines.

As for the methods, private counseling and interaction through social media were novel approach that has not been tested in Indonesia. While promotion through physical media (e.g. poster, leaflet, and magazine) were no longer recommended by the experts. In terms of human resources, the leaders of the workplace hold extremely important role. Several studies showed that the leader’s attitude would reflect on the policy/regulation outcomes. Providing lactation counselor at workplace would be costly for the management, however, it might reduce the employee’s absence for taking care of their sick children, which could also lessen the productivity cost.

This study is using an online approach for three-round Delphi surveys. The respondents did not know each other and hence ensure the result to be independent and free of peer pressure and influence from other experts. This method was also relatively less expensive than face-to-face meeting. However, this method was time consuming and required intensive approach to the respondents. The time gap between first and second round was relatively long, around one month while between second and the last round was shorter. Since it took a long duration, the respondents should be committed in joining this study.

It should be noted as well that Delphi approach aims for a consensus; and not pointed out which of the answers were correct. There might be a risk that certain aspects were not included. This would be depending to the selection of experts. In this study, we involved various experts, from clinical expertise and policy makers. However, we did not involve the target of the module, i.e. working mothers and employers. Further studies would be necessary to assess the effectiveness of the module.

## Conclusions

In the views of experts, a top-down approach involving the policy makers in the company, a workplace-based lactation counselor, and regular promotion method with interactive education and dedicated facilities are necessary to develop an effective lactation program at work.

## Abbreviations

Q: Quartile
SSPS: Statistical Package for the Social Sciences
UNICEF: United Nations Children’s Fund
WHO: World Health Organization
